# Aromatic Amino Acid Hydroxylases as Off-Targets of Histone Deacetylase Inhibitors

**DOI:** 10.1021/acschemneuro.4c00346

**Published:** 2024-11-11

**Authors:** Anne Baumann, Niklas Papenkordt, Dina Robaa, Peter D. Szigetvari, Anja Vogelmann, Franz Bracher, Wolfgang Sippl, Manfred Jung, Jan Haavik

**Affiliations:** † Department of Biomedicine, University of Bergen, 5007 Bergen, Norway; ‡ Institute of Pharmaceutical Sciences, University of Freiburg, 79104 Freiburg, Germany; § Institute of Pharmacy, Martin-Luther University of Halle – Wittenberg, 06120 Halle/Saale, Germany; ∥ Division of Psychiatry, Haukeland University Hospital, 5009 Bergen, Norway; ⊥ Department of Pharmacy – Center for Drug Research, Ludwig-Maximilians University Munich, 81377 Munich, Germany; # Bergen Center for Brain Plasticity, Division of Psychiatry, Haukeland University Hospital, 5009 Bergen, Norway

**Keywords:** histone deacetylase, tryptophan hydroxylase, phenylalanine hydroxylase, tyrosine hydroxylase, iron chelators, epigenetics

## Abstract

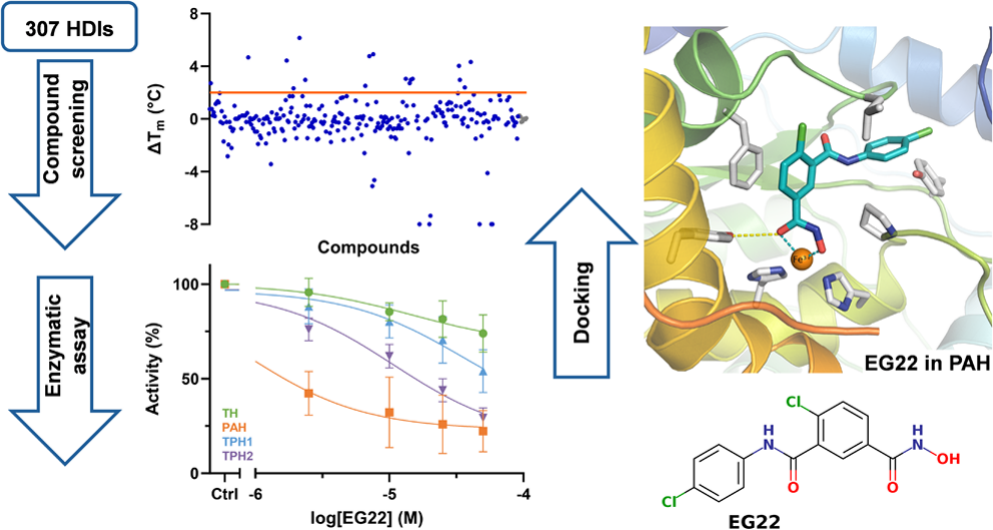

The aromatic amino acid hydroxylases (AAAHs) phenylalanine hydroxylase, tyrosine hydroxylase, and tryptophan hydroxylases 1 and 2 are structurally related enzymes that contain an active site iron atom and depend on tetrahydrobiopterin (BH_4_) as cosubstrate. Due to their important roles in synthesis of serotonin, dopamine, noradrenaline, and adrenaline and their involvement in cardiovascular, neurological, and endocrine disorders, AAAHs have been targeted by substrate analogs, iron chelators, and allosteric ligands. Phenylalanine hydroxylase is also off-target of the histone deacetylase (HDAC) inhibitor panobinostat. To systematically explore the binding of HDAC inhibitors to AAAHs, we screened a library of 307 HDAC inhibitors and structural analogs against tryptophan hydroxylase 1 using a fluorescence-based thermal stability assay, followed by activity assays. Selected hits were enzymatically tested against all four purified human AAAHs. Cellular thermal shift assay was performed for phenylalanine hydroxylase. We show that panobinostat and structurally related compounds such as TB57, which similarly to panobinostat also contains a cinnamoyl hydroxamate, bind to human AAAHs and inhibit these enzymes with high selectivity within the class (panobinostat inhibition (IC_50_): phenylalanine hydroxylase (18 nM) > tyrosine hydroxylase (450 nM) > tryptophan hydroxylase 1 (1960 nM). This study shows that panobinostat and related hydroxamic acid type HDAC inhibitors inhibit all AAAHs at therapeutically relevant concentrations. Our results warrant further investigations of the off-target relevance of HDAC inhibitors intended for clinical use and provide directions for new dual HDAC/AAAH and selective AAAH inhibitors. These findings may also provide a new mechanistic link between regulation of histone modification, AAAH function, and monoaminergic neurotransmission.

## Introduction

The aromatic amino acid hydroxylases (AAAHs) phenylalanine hydroxylase (PAH), tyrosine hydroxylase (TH), and tryptophan hydroxylase (TPH) are structurally related enzymes that all contain an active site ferrous ion and depend on a tetrahydrobiopterin (BH_4_) cosubstrate as electron donor.^1^

TH and TPH catalyze the rate limiting steps in the biosynthesis of catecholamines and serotonin, respectively, while PAH is responsible for converting phenylalanine into tyrosine that is used in protein biosynthesis, converted into thyroid hormones, or further metabolized. TPH exists as two structurally related enzymes (TPH1 and TPH2) encoded by different genes and expressed in different tissues; TPH1 is mainly found in enterochromaffin cells of the intestines, but also in adrenal glands, kidney, and the pineal gland, and TPH2 in serotonergic neurons.^2^ Human TH is encoded by a single gene on chromosome 11 but is subject to alternative splicing, generating four isoforms with slightly different N-terminal sequences (TH1–TH4), where TH1 is the shortest and most abundant isoform.^3,4^

Due to the important physiological roles of serotonin, dopamine, noradrenaline, and adrenaline and their involvement in cardiovascular, neurological, and endocrine disorders, monoamine-related enzymes, receptors, and transporters are important therapeutic targets. This also includes the AAAHs, which have been targeted by amino acid and tetrahydrobiopterin analogues, iron chelators, and allosteric ligands.

Since loss of function variants in the liver enzyme PAH are the most common causes of phenylketonuria, attempts have been made to design pharmacological chaperones that can correct misfolded protein variants of PAH.^5^ TH inhibitors decrease the production of catecholamines and have been tested as antihypertensives^6^ and in neurological/neuropsychiatric disorders, including infantile spasms^7^ and autism.^8^

Multiple TPH inhibitors have been developed and have been subjected to clinical trials. First synthesized and tested in the 1950s, the substrate analogue 4-chloro-l-phenylalanine (L-PCPA or fenclonine) has since been widely used in animal research and in patients with carcinoid syndrome and emesis induced by chemotherapy.^9^ Lexicon Pharmaceuticals used L-PCPA as a starting point to develop more potent TPH inhibitors, such as LP-521834, LP-534193, and LP-533401.^9^ Telotristat ethyl, via its active metabolite Telotristat (LX-1032, LX-1606, and LP-778914), provides symptomatic relief in patients with carcinoid syndrome and is approved by the US FDA.^10^ More recently, a series of spirocyclic, proline-based TPH1 inhibitors, such as KAR5585 and KAR5417, have been developed by Karos Pharmaceuticals^11^ and are being tested in patients with pulmonary arterial hypertension (ELEVATE 2 (NCT04712669) trial).^12^ Similarly, the novel TPH inhibitor TPT-004^13,14^ has shown promising results in a rat model of pulmonary arterial hypertension.^15^

TPH inhibitors belong to various chemical classes and have different selectivities and modes of action. Screening of libraries of established drugs has identified several compounds that showed nanomolar affinity for TPH and TPH2 and inhibitory or stabilizing effects of these enzymes, raising the possibility that previously known drugs may be repurposed as AAAH inhibitors or activators.^16−18^ Allosteric inhibitors of TPH1 have also been reported.^19^

Using a proteome profiling strategy, it was recently shown that PAH and TH are off-targets of the nonselective histone deacetylase (HDAC) inhibitor (HDI) panobinostat, with IC_50_ values of 0.2 and 3.3 μM, respectively.^20^ For comparison, the half-maximal inhibitory concentration for this pan HDAC inhibitor ranges between 2.1 and 531 nM against HDACs across classes. Based on the binding profiles, it was suggested that the AAAH inhibition is related to the presence of the *N*-hydroxycinnamide warhead of panobinostat but no further data on this hypothesis was provided.^20^ HDIs target histone deacetylases, a large class of epigenetic regulators that are established treatment targets in cancer, and promising targets in other conditions such as virus infections, inflammatory, neuropsychiatric and neurodegenerative diseases.^21^

To test whether AAAHs are targeted by other HDIs and explore their binding selectivity, we screened an in-house library of 307 HDAC inhibitors and structural analogs against human TPH1. Compounds that showed stabilizing effects and had more than 50% enzyme inhibition were subjected to dose–response testing against PAH, TH, and TPH2. We show that panobinostat and structurally related compounds such as TB57 and EG22, which are also cinnamoyl hydroxamates, bind to human TH, PAH, and TPH and inhibit these enzymes at low micromolar concentrations. Binding of these inhibitors to PAH in intact cells was validated by using a cellular thermal shift assay (CETSA). Our results highlight panobinostat and structurally related compounds as promising new AAAH inhibitors worthy of further development.

## Results and Discussion

### Effects of HDIs on Thermal Stability of AAAHs

**Figure 1:**
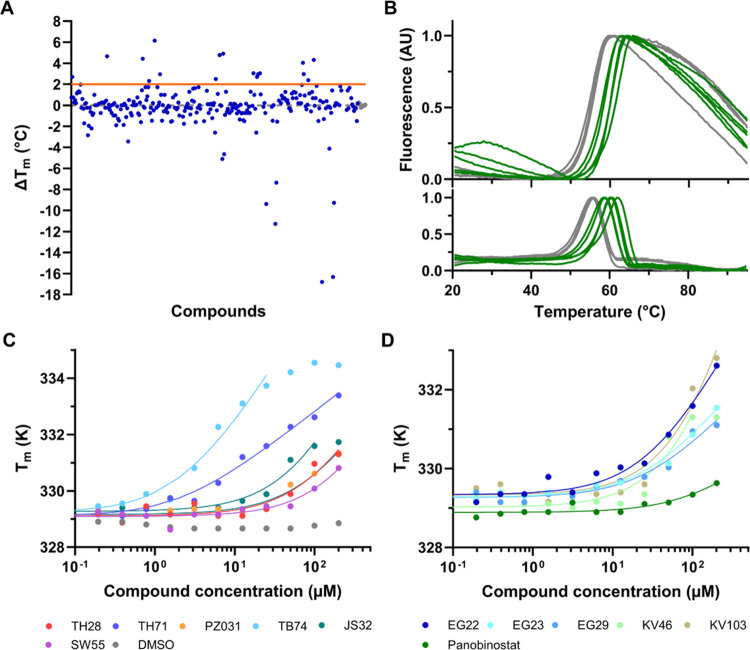
Representative results from the HTS against TPH1 using DSF and the effect of increasing compound concentration on the *T*_m_ of TPH1. (A) Graph shows Δ*T*_m_ plotted versus compounds (blue dots). DMSO-only controls are shown in gray. The orange line shows the cut-off at Δ*T*_m_ = +2 °C. (B) Thermal melting curves from primary screening were monitored by DSF at 0.075 mg/mL (2.1 μM) TPH1 in 0.5% DMSO (upper trace) and the first derivative (bottom trace) with representative stabilizers (green) at 50 μM. Gray curves are 0.5% DMSO controls. (C, D) Among the initial 15 hits considered, the 12 compounds shown in panel C and D induced a concentration-dependent shift of the *T*_m_ of TPH1. DMSO control is shown in gray. For data fitting, see materials and methods. Some compounds (e.g., TB74, JS32, and KV46) had solubility issues at the highest concentrations. The highest concentrations of these compounds were removed before curve fitting. The chemical name, structure, and molecular weight of the selected compounds are shown in Table S1, while comparative subtype selectivities against HDACs and AAAHs for the selected compounds contained in Table S3 were compiled from the literature, as well as this study. A positive control panobinostat^20^ and reference HDIs (mocetinostat, entinostat, and vorinostat) are shown together with DMSO (solvent control) in Figure S2.

High throughput screening (HTS) using a fluorescence-based thermal stability assay (differential scanning fluorimetry, DSF) showed that 15 out of 307 (including 3 controls; mocetinostat, entinostat and vorinostat) HDIs and structural analogs tested had a clear (>+2 °C) stabilizing effect on TPH1 (Figure 1A,B). Some HDIs showed a destabilizing effect on TPH1, but these compounds were not subjected to further analysis. A dose–response effect was established for the 15 most potent stabilizing compounds using serial dilution of the compounds from 200 μM down to 98 nM, keeping the DMSO concentration constant at 2% (Figure 1C,D). As shown in Table 1, the apparent *K*_D_ estimated from DSF data varied from 1.4 μM for TB74 to >100 μM for several other HDIs.

**Table 1:** *T*_m_, Δ*T*_m_, and Apparent *K*_D_ (from Dose–Response Experiments) of Selected Hits from the Primary Screen (*T*_m_ and Δ*T*_m_ are Calculated from Three Independent Measurements)

	primary screen		dose-response	
	*T*_m_ (°C) ± SD (°C)	Δ*T*_m_ (°C) ± SD (°C)	apparent *K*_D_ (μM)	*R^2^*
TH137A	56.7 ± 2.4	1.3 ± 2.6		
PZ031	57.0 ± 0.2	1.6 ± 0.1	54.6	0.86
2-(2-OH-phenyl)benzoxazol	57.6 ± 0.7	2.3 ± 0.9		
EG29	57.8 ± 0.0	2.3 ± 0.2	10.3	0.88
SW189	58.0 ± 0.3	2.7 ± 0.0		
TH28	58.1 ± 0.3	2.8 ± 0.1	76.3	0.84
EG23	58.2 ± 0.2	2.7 ± 0.2	14.1	0.95
SW55	58.4 ± 0.3^a^	3.0 ± 0.1	87.2	0.79
JS32	58.5 ± 0.2^a^	3.1 ± 0.0	30.9^b^	0.90
KV46	59.1 ± 0.5^a^	3.6 ± 0.3	>100.0^c^	0.82
EG22	59.4 ± 0.3	3.9 ± 0.2	15.2	0.96
KV103	59.6 ± 0.7	4.3 ± 0.5	49.4	0.90
TB74	59.7 ± 0.4	4.4 ± 0.4	1.4^d^	0.96
TH71	60.0 ± 0.3	4.7 ± 0.1	1.8	0.97
panobinostat	56.0 ± 0.0	0.4 ± 0.0	15.6	0.79
mocetinostat	54.5 ± 0.2	–0.8 ± 0.1		
vorinostat	56.4 ± 1.9	1.1 ± 2.1		
entinostat	56.0 ± 0.4	0.7 ± 0.3		
DMSO	55.3 ± 0.3			
DMSO	55.4 ± 0.2			

a Duplicates.

b Removed 200 μM data point for curve fitting.

c Removed 200 and 0.391 μM point for curve fitting.

d Removed 200, 100 and 50 μM point for curve fitting.

**Figure 2:**
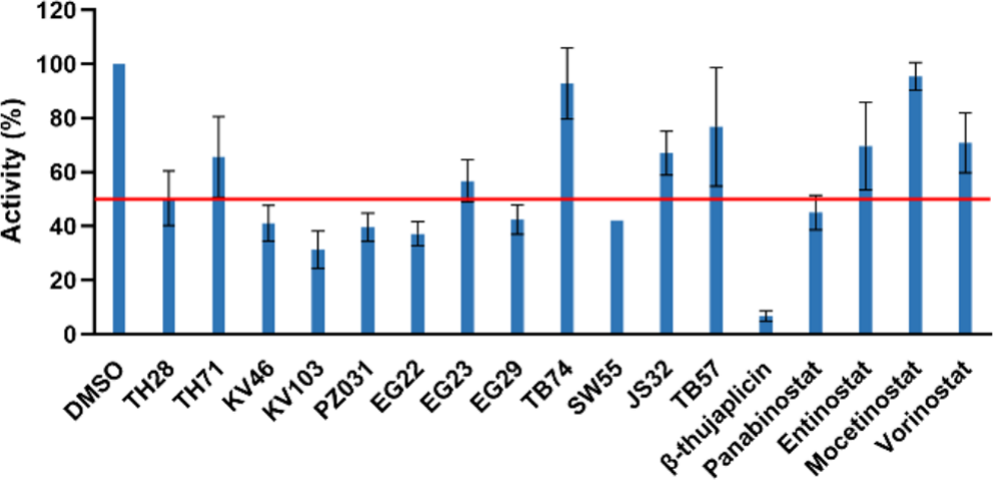
Effect of selected hits from the HTS on the TPH1 activity. Remaining TPH1 activity in the presence of 17 compounds (including the three negative controls from the DSF screen and β-thujaplicin as well as TB57) (compound concentration 50 μM; *n* = 2; mean ± SD). The red line shows the 50% limit that was set as a threshold for further characterization.

Next, we tested the effects of these 12 compounds on the enzyme activity of human TPH1 (Figure 2). β-Thujaplicin was also examined since it represents a different chemical class, despite showing a destabilizing effect on TPH1 (see Figures S1 and S2). DMSO itself (0.5%) reduced the activity of TPH1 by 3.2 ± 7.4% (mean ± SD, *n* = 36 all control samples in this work).

**Figure 3:**
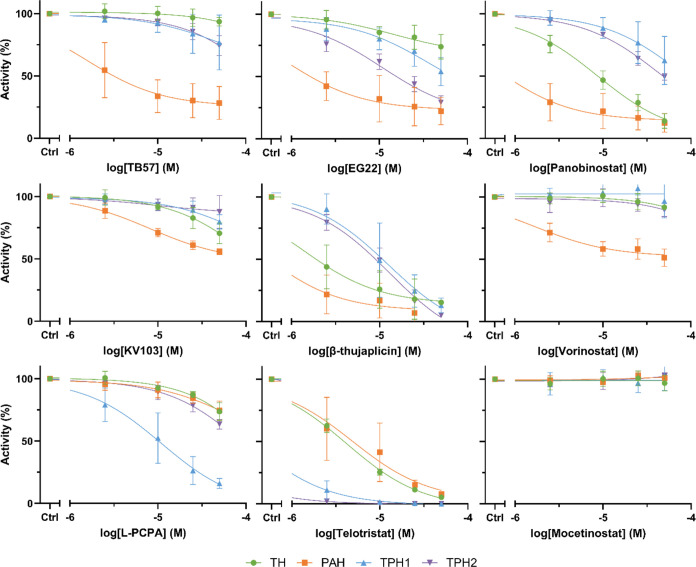
Activity of TPH1, TPH2, PAH, and TH in the presence of selected hits. An estimate of the IC_50_ was acquired by nonlinear regression curve fitting. The data are expressed as means ± SD from two independent experiments, each performed in duplicate (except TPH1 in the presence of panobinostat: four independent experiments, each performed in duplicate).

The compounds that showed more than 50% inhibition at 50 μM (panobinostat, EG22, β-thujaplicin, KV103, as well as the reference inhibitors telotristat, mocetinostat, L-PCPA, vorinostat, and the structural analogue TB57), were then screened in dose–response experiments for all four AAAHs (Figure 3).

As shown in Table 2, several HDIs inhibited the AAAHs at low micromolar concentrations. However, there were obvious differences between the different enzymes. In general, PAH appeared to be most sensitive toward inhibition, with IC_50_ values of 0.5 and 0.9 μM for β-thujaplicin and EG22, respectively, while TPH1 and TPH2 were least affected.

**Table 2:** Effect of Inhibitory Hits on TH, PAH, THP1, and TPH2 Activity

	IC_50_ (μM)^a^			
compound	TH	PAH	TPH1	TPH2
panobinostat	8.7 (±1.7)	0.6 (±0.3)	95.4 (±74.8)	43.6 (±11.5)
TB57		1.6 (±0.8)	49.6 (±117.9)	194.7 (±401.1)
EG22	14.7 (±12.2)	0.9 (±0.5)	35.8 (±31.0)	10.7 (±2.9)
β-thujaplicin	1.4 (±0.6)	0.5 (±0.3)	12.6 (±6.0)	12.9 (±2.6)
telotristat	4.2 (±0.3)	4.9 (±2.6)	3.4 × 10^–1^ (±6.6 × 10^–2^)	5.5 × 10^–2^ (±1.0 × 10^–2^)

a Best-fit IC_50_ (± SE) values obtained using nonlinear regression curve fitting.

A comparison of binding affinities as determined by DSF and inhibitory activity against the AAAHs showed little correlation between protein binding and inhibitory activity (Table 1 and Figure 3). For example, TB74 which showed high affinity binding using DSF (apparent *K*_D_ = 0.7 μM) showed no significant inhibition at 50 μM and β-thujaplicin, which had very low affinity as determined by DSF, had an estimated IC_50_ of 0.5 μM in the activity assay. This potentially indicates the presence of two different binding modes, an active site binding, presumably interacting with the active site iron, and another allosteric binding site. Mocetinostat, which is an *N*-(2-aminophenyl)-benzamide type zinc-binding HDI was completely inactive as an inhibitor against all the AAAHs (Figures 3 and S3).

**Figure 4:**
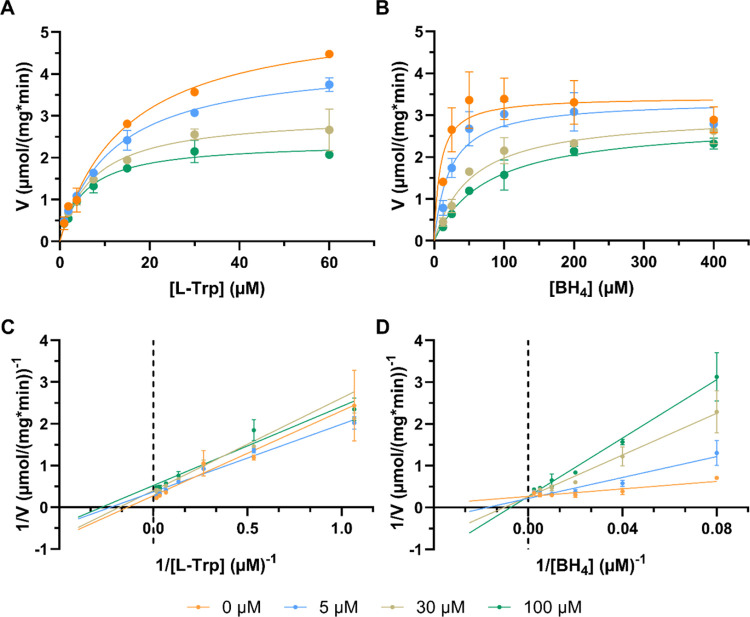
Kinetic study of TPH1 inhibition by EG22. (A, B) Michaelis–Menten equation was fitted to data using nonlinear regression. (C, D) Lineweaver–Burk plots of reaction velocity versus substrate concentration for enzyme kinetics of TPH1 at varying concentrations of EG22 (0–100 μM). The data are expressed as the means ± SD from two independent experiments. The resulting steady-state kinetic parameters are shown in Table 3.

TPH1 and TH were used for kinetic protein–ligand interaction studies. The mechanism of inhibition was investigated by measuring enzyme inhibition at different concentrations of the inhibitor, amino acid substrate, and BH_4_ cofactor. Kinetic parameters, *K*_m_ and *V*_max_, were extracted from Michaelis–Menten curve fitting (Figures 4A,B and S4 and Tables 3 and S2). Lineweaver–Burk plots for TPH1 were obtained for both l-Trp and BH_4_ by plotting the reciprocal rate of the enzyme reaction (1/*V*) versus the reciprocal substrate concentration (1/[*S*]) in the absence and presence of EG22 (0–100 μM) (Figure 4C,D). At varying l-Trp and fixed BH_4_ concentration a decrease in *V*_max_ and *K*_m_ was observed with increasing inhibitor concentration suggesting an apparent mixed/uncompetitive inhibition. When the concentration of BH_4_ was varied in the presence of a fixed l-Trp concentration an increase in *K*_m_ and almost unchanged *V*_max_ was detected, consistent with a competitive inhibition. Kinetic parameters for TH in the presence of panobinostat also showed a mixed competitive/noncompetitive inhibition toward BH_4_ (Figure S4 and Table S2).

**Table 3:** TPH1 Kinetic Parameters in the Presence of EG22^a^

	l-Trp 0.937–60 μM/BH_4_ 200 μM		BH_4_ 12.5–400 μM/l-Trp 30 μM	
EG22 (μM)	*V*_max_ (μmol/(min·mg))	*K*_m_ (μM)	*V*_max_ (μmol/(min·mg))	*K*_m_ (μM)
0	5.5 ± 0.2	14.6 ± 1.8	3.4 ± 0.3	8.5 ± 4.7
5	4.4 ± 0.2	11.7 ± 1.1	3.4 ± 0.2	22.3 ± 6.8
30	3.0 ± 0.2	7.5 ± 1.3	3.0 ± 0.2	53.8 ± 11.2
100	2.4 ± 0.1	5.5 ± 0.8	2.8 ± 0.2	76.9 ± 12.5

a Data represent means of two independent experiments, and best-fit *V*_max_ (± SE) and *K*_m_ (± SE) values were extracted from Michaelis−Menten nonlinear regression curve fitting.

**Figure 5:**
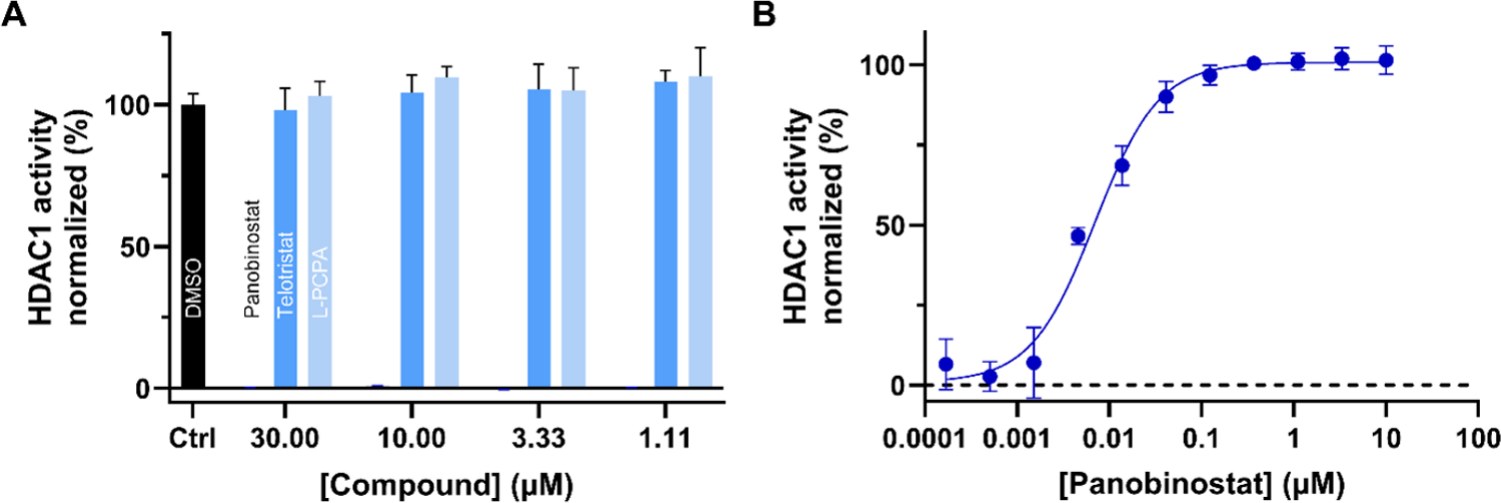
HDAC1 activity in response to panobinostat and two classical AAAH inhibitors (telotristat and l-PCPA). (A) Normalized HDAC1 activity was measured in the presence of varying concentrations (1.11–30 μM) of panobinostat, telotristat, and l-PCPA, with DMSO (5%) serving as a control. The bar for panobinostat is barely visible due to the strong inhibition at these concentrations. (B) Panobinostat inhibits HDAC1 at low concentrations in a concentration-dependent manner, with an IC_50_ value of 7.0 ± 0.8 nM. All data are expressed as means ± SD from triplicates.

The HDAC1 activity of telotristat and l-PCPA, two classical AAAH inhibitors, was evaluated alongside that of panobinostat. The results are presented in Figure 5. Findings indicate that telotristat and l-PCPA do not inhibit the HDAC1 activity. In contrast, panobinostat inhibits HDAC1 activity in a concentration-dependent manner, with an IC_50_ value of 7.0 ± 0.8 nM.

### Cellular Thermal Shift Assay (CETSA) in HepG2 Cells

**Figure 6:**
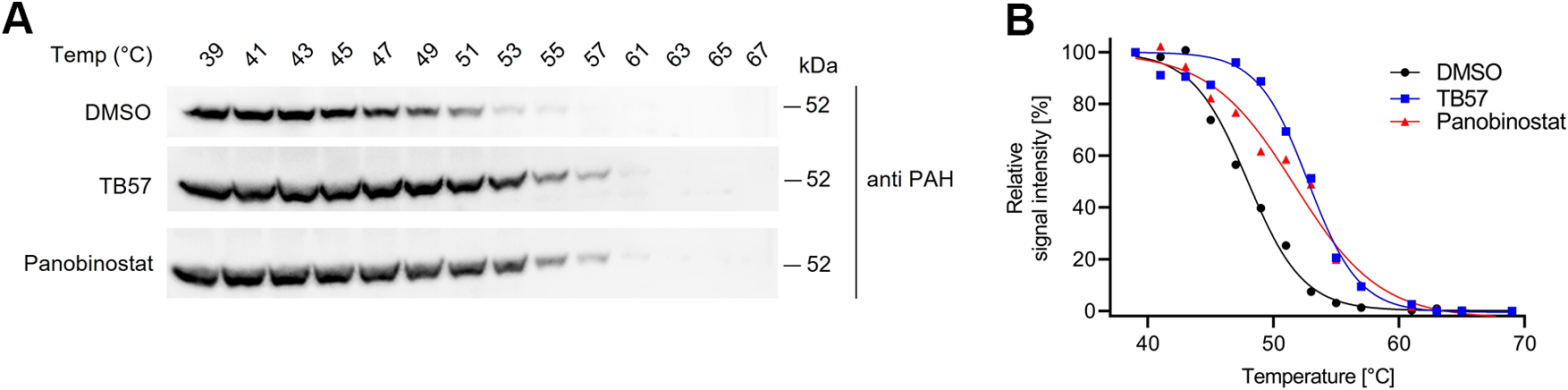
CETSA for PAH in HepG2 cells treated with vehicle (DMSO), 50 μM TB57 or panobinostat. Representative Western Blots (A) and quantification (B) showing increased melting temperatures (Δ*T*_m_) of endogenous PAH upon treatment with TB57 or panobinostat compared to DMSO.

The outcomes from the *in vitro* screening revealed TB57 as the sole compound exhibiting no inhibition of TH, while demonstrating selectivity toward PAH in comparison to TPH1 (30-fold) and TPH2 (125-fold) (Table 2). To assess cellular target engagement of TB57, cellular thermal shift assays (CETSA) were performed in the human hepatoblastoma cell line HepG2, which revealed stronger stabilization of endogenous PAH (Δ*T*_m_ = 4.8 °C) compared to panobinostat (3.7 °C) or DMSO (Figure 6).

### Ligand Docking of TB57 and EG22 into PAH, TPH1, and TH

**Figure 7:**
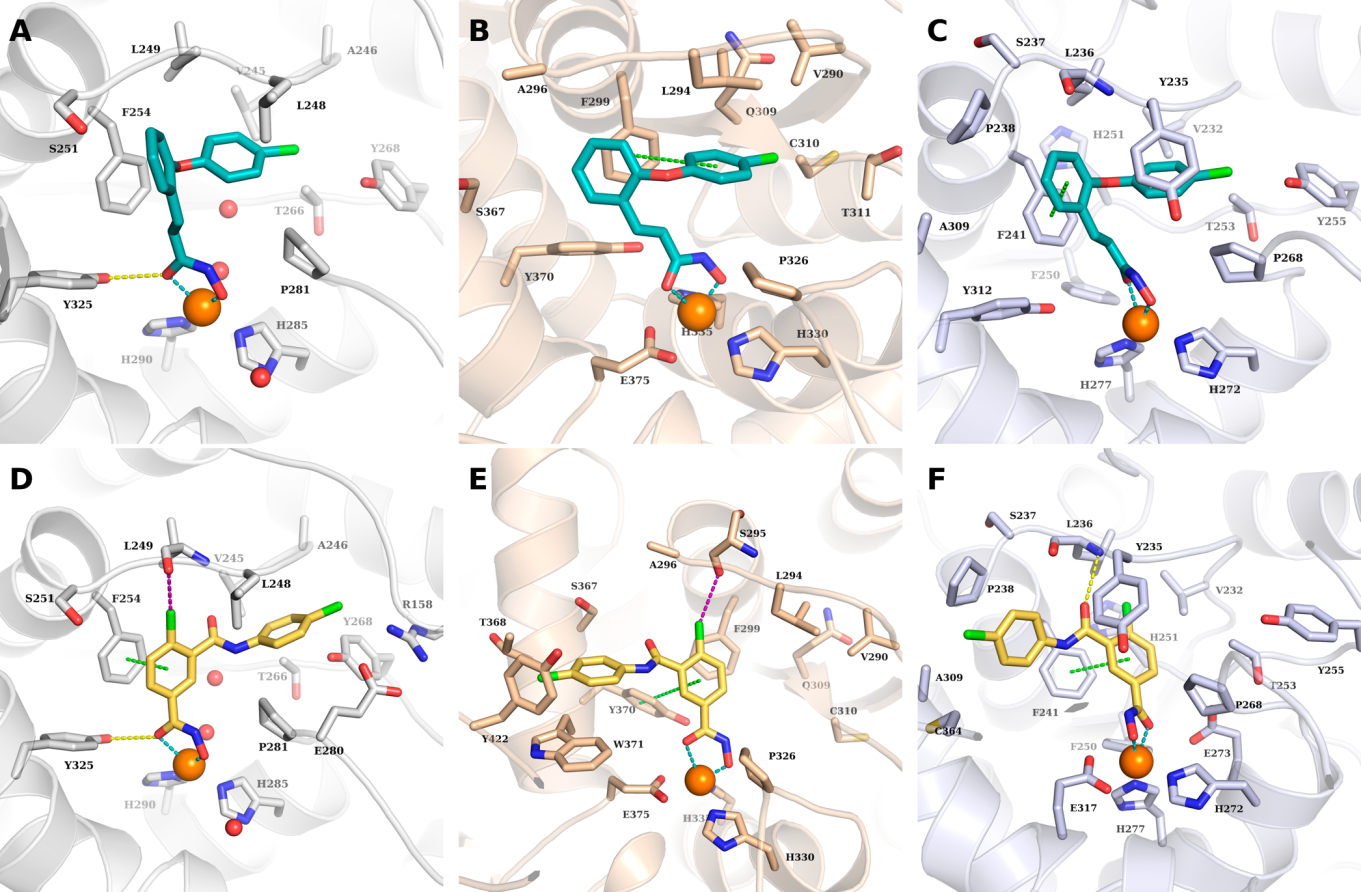
Predicted binding mode of TB57 (teal sticks) in (A) PAH (white; PDB ID: 3PAH), (B) TH (beige; PDB ID: 6ZN2), and (C) TPH1 (light blue; PDB ID: 8CJM) and predicted binding mode of EG22 (yellow sticks) in (D) PAH (white; PDB ID: 3PAH), (E) TH (beige; PDB ID: 6ZN2), and (F) TPH1 (light blue; PDB ID: 8CJM). The Fe(II) ion is shown as orange sphere, and the water molecules as red spheres. Metal coordination is shown as dashed teal lines; hydrogen bonds as yellow-dashed lines, π–π interactions as green-dashed lines, and halogen bonds as purple-dashed lines.

Docking studies were conducted into available crystal structures of PAH (PDB ID: 3PAH), TPH1 (PDB ID: 8CJM), and TH (PDB ID: 6ZN2). The obtained docking poses of TB57 show similar predicted binding conformations in the binding pocket of the three enzymes (Figure 7). The hydroxamate group chelates the catalytic iron(II) ion in a bidentate manner. In the case of PAH, an additional hydrogen bond interaction could be predicted between the hydroxamate moiety and the side chain of Tyr325. The phenylethylene linker is placed near the aromatic side chain of the conserved Phe254 where hydrophobic interactions could be observed. In TPH1, an additional π–π stacking interaction is formed between the phenyl group of the linker and the side chain of Phe254. The aromatic capping group is embedded in a hydrophobic pocket where it undergoes hydrophobic interactions with four conserved hydrophobic amino acid residues (Pro281/326/268, Thr266/311/253, Leu249/294/236, and Val245/290/232 in PAH, TH and TPH1; respectively) in addition to one nonconserved residue, namely, Leu248 in PAH and Tyr235 in TPH1.

The docking poses of EG22 in PAH (PDB ID: 3PAH) showed a similar predicted binding mode to that obtained for TB57 (Figure 7A). The benzhydroxamate moiety undergoes a hydrogen bond interaction with the side chain of Tyr325 and an π–π stacking interaction with the side chain of Phe254. An additional halogen bond could be observed between the chloro substituent and the backbone-carbonyl group of Leu249. The terminal phenyl group is embedded in the same hydrophobic region as that of TB57. Meanwhile, a different binding mode was obtained for EG22 in TPH1 and TH. The hydroxamate moiety is still able to chelate the iron(II) ion in a bidentate fashion, while the central phenyl group shows π–π interactions with the conserved Phe241 residue in TPH1 or Tyr 370 in TH. Meanwhile the capping group is embedded in an opposite binding pocket, where it undergoes hydrophobic interactions with the surrounding residues, Trp375, Tyr422, and Thr368 in TH, and Pro238, Cys364, and A309 in TPH1.

### Effects of HDIs on Enzyme Activity

Class I and II (classical) HDACs contain an active site zinc(II) ion, which is required for their catalytic action. Class III HDACs (Sirt1-7) do not contain metal ions but are nicotinamide adenine dinucleotide (NAD^+^)-dependent enzymes. In contrast, the AAAHs all contain an active site nonheme iron atom, which is necessary for their oxygen activation and catalytic turnover. Although the enzymes are only active in the presence of Fe(II), AAAHs can bind other divalent transition metal ions as well as Fe(III). At pH 6.5, the affinity of zinc(II) for TH is 3–5 times higher than for iron(II)^22^ and TH isolated from mammalian tissues contain significant amounts of zinc, in addition to iron.^23^

Due to its high affinity to zinc(II), hydroxamic acid is the most used metal coordinating moiety among HDIs.^21,24^ Hydroxamates are also potent iron(III) chelators and hydroxamates are inhibiting TPH1, PAH, and other iron containing enzymes.^25,26^ For instance, deferoxamine is a microbial siderophore with three bidentate hydroxamate functional groups with very high affinity for Fe(III) and some affinity for Fe(II).^27^ Among the HDIs tested here, β-thujaplicin was among the most potent inhibitors of all AAAHs, with IC_50_ values from 0.5 to 12.9 μM (Tables 2 and S3). Thujaplicins and other hydroxytropolones are potent nonselective metal ion chelators, forming complexes with Fe(II), Cu(II), and Zn(II) as also observed for catecholate complexes with such transition metals. In addition to targeting HDACs,^28^ thujaplicins have antioxidant properties that may be related to their inhibition of the copper containing enzymes tyrosinase and polyphenol oxidase.^29^

The active site iron in AAAHs can be targeted by many different iron chelators that can either extract iron from the active site or bind tightly to the active site Fe(II), alternatively sequester the iron in the inactive Fe(III) state and block its interaction with the BH_4_ cosubstrate, oxygen, and amino acid substrates. Ethylenediamine, EDTA, dipicolinic acid, and 1,10-phenanthroline (o-phenanthroline) extract iron from the active site of TH, while catechol and the catechol derivatives dopamine, noradrenaline, and apomorphine form stable complexes with Fe(III) in the active site.^22,30^ The tight binding of catecholamines to active site iron explains the blue-green color of pure TH isolated from adrenal medulla.^23^ Such stable complexes between Fe(III) and catecholates are also found in nature, including the byssal threads of marine mussels^31^ and have recently been used to produce potent adhesives and glues for medical and industrial purposes.^32^

Negatively charged metal chelators such as bathophenanthroline sulfonate, 4,5-dihydroxybenzene-1,3-disulfonate (Tiron), and bathocuproine sulfonate have less inhibitory effect than 1,10-phenanthroline, possibly due to their relative preference for Fe(III), Cu(II), and limited access to the active site iron.^22,23^

Based on these considerations, we suggest that the inhibitory effects of the HDIs tested here are mainly related to their ability to bind to the active site iron in the AAAHs. The differences in inhibitory action of the HDIs may be related to their size, charge, and access to the iron atom, as well as their relative preference for either Fe(II) or Fe(III). The inhibitory effect of HDIs on AAAHs was observed across several chemical classes of HDIs, with no obvious correlation between HDAC specificity and hydroxylase inhibitor selectivity (Table S3). It seems that a strong chelating group like a hydroxamate (or α-hydroxyketone like in β-thujaplicin) is necessary but not sufficient for inhibition, but the lack of inhibition among *N*-(2-aminophenyl)-benzamide inhibitors like mocetinostat could also be due to the fact that there is a steric clash in the active site. Hybrids of e.g., TB57 or EG22 with other zinc-chelating warheads could test that hypothesis. In addition to AAAHs, other iron or zinc containing enzymes may also be inhibited by metal chelating HDIs. However, the extent of inhibition depends on the particular structure of the ligands and enzyme active sites. Out of a series of hydroxamate-based HDAC inhibitors only one specific compound blocked Zn-dependent matrix metalloproteases, while the others were selective.^33^ The kinetic mechanism of inhibitor binding to AAAHs has previously been explored for metal chelating and other types of inhibitors. Our steady-state kinetic studies on TPH1 and TH showed that EG22 and panobinostat were mainly competitive inhibitors versus BH_4_ and mixed noncompetitive/uncompetitive versus the amino acid substrates, as also observed for the panabinostat inhibition of PAH^20^ and other metal chelating TH and TPH inhibitors.^34−36^

## Biological Implications

During the past 20 years, HDIs have been used for multiple clinical indications within oncology, neurology, and against inflammatory and infectious diseases.^21,37^ HDIs belong to different chemical classes with distinct modes of action, variable target affinities, subtype selectivity, and off-target effects.^20,24,38^ Although primarily designed to target HDACs, HDIs can affect gene expression and epigenetic modifications not only by inhibiting HDACs, but also through altering acetylation of transcription factors and other proteins.^38,39^ Here we show that all four human AAAHs are inhibited at micromolar concentrations of HDIs, but only PAH and TH are inhibited at therapeutically relevant compound concentrations. EG22 mainly inhibited PAH and TPH2, while panobinostat and β-thujaplicin were most active against TH and TPH (Figure 3 and Table S3). HDI binding was tested for purified human enzymes, target engagement was verified for PAH expressed in a hepatoblastoma cell line, the relative inhibitory activity was established using purified PAH, TH1, TPH1, and TPH2 and ligand docking showed that different HDIs can interact with the active site ferrous iron and adopt a similar binding conformation in the binding pocket of these enzymes.

As serotonin is involved in many physiological functions, TPH1 or TPH2 inhibitors, in particular, might have a wide range of clinical applications. TPH1 inhibitors decrease levels of serotonin in the periphery and increase bone formation,^40^ although the effectiveness of TPH1 inhibitors in osteoporosis has been debated.^41^ During the past decades, TPH inhibitors have also been tested in other conditions, such as carcinoid syndrome, ulcerative colitis, irritable bowel disease, obesity, asthma, lung fibrosis, and pulmonary arterial hypertension.^15,42^ Allosteric activators and stabilizers of TPH2 have also been reported.^18^ Similar to TPH1 and TPH2, TH activity is tightly regulated at the transcription levels as well as by posttranslational mechanisms. As TH and TPH are rate limiting, regulatory enzymes involved in the synthesis of catecholamines and serotonin, respectively, the direct inhibition of these enzymes at therapeutic levels of HDIs could be of clinical relevance.

HDACs have been implicated in the regulation of key proteins involved in monoamine signaling, including TPH1, TPH2, TH, and the monoamine transporters SERT, DAT, and NET.^43,44^ Different HDIs can also affect many enzymes that are not involved in protein acetylation/deacetylation, and in many instances these off-target effects can be the major mechanisms of action.^45^ It is possible that HDIs could affect serotonin and catecholamine functions through multiple mechanisms. Treatment of neuroblastoma cells with the hydroxamate-type HDI trichostatin A increased the expression of the dopamine and serotonin transporters 45- and 15-fold, respectively.^43^ As hydroxamate-type HDIs may also be direct inhibitors of TH, TPH1, and TPH2 (Figure 4), it is conceivable that an upregulation of monoamine transporter activity combined with the inhibition of TH and TPH could both contribute to decreased extracellular levels of monoamines. Thus, HDAC/AAAH dual inhibitors could have therapeutic potential in cases where the aim is to reduce extracellular levels of monoamines.

Regulation of TH and TPH2 is important in presynaptic neuronal plasticity. Epigenetic regulation of serotonergic neurotransmission is believed to mediate the therapeutic effects and neuroplasticity induced by selective serotonin reuptake type of antidepressants (SSRIs).^46^ Recently, it has been reported that dopamine and serotonin also bind covalently to histones and affect gene transcription in peripheral and brain tissues, similar to the effects of histone acetylation on these proteins.^47^ Thus, inhibitors of TH and TPH1/TPH2 might affect histone modification and monoaminergic functions by multiple mechanisms.

In conclusion, our studies suggest that clinical side effects associated with HDIs may originate from transcriptional effects on neurotransmission as well as concurrent or concomitant direct inhibition of aromatic amino acid hydroxylases. Moreover, our data provide a basis for the development of dual HDAC/amino acid hydroxylase inhibitors or, as it is easy to abolish the HDAC inhibitory activity of hydroxamates, e.g., by *N*-methylation, can provide a basis for new selective hydroxylase inhibitors (Figure S5). Such an *N*-methylation abolished HDAC inhibition and did lead to selective inhibition of iron dependent histone demethylases proving the feasibility of this approach.^48^

## Methods

### HDAC Inhibitors

We assembled an in-house library of HDIs from different inhibitor projects from our groups representing HDIs from different chemical classes and HDAC specificities. Compounds were dissolved in DMSO in 10 mM stocks.

### Enzymes

Full-length human TH isoform 1 (TH1), human PAH, human TPH2, and doubly truncated human TPH1 (ΔNH102-ΔCOOH402) were expressed in *E. coli* and purified as described.^16,22,49−51^

The ΔNH102-ΔCOOH402 human TPH1 gene was cloned into the pET23a vector (His_6_ C-terminal fusion) (Novagen) between restriction sites NdeI and XhoI and overexpressed using BL21(DE3) cells in LB medium with 100 μg/mL ampicillin and 0.2 mM ferrous ammonium sulfate at 37 °C to an OD_600_ of ∼0.8, with 1 mM IPTG induction at 25 °C for 15 h. The cell pellet was lysed in buffer (50 mM Tris at pH 8.0, 400 mM NaCl, 3 mM methionine, 1 mM MgCl_2_, 5% glycerol, benzonase, EDTA-free protease inhibitor cocktail (Complete EDTA-free; Roche Diagnostics GmbH), and 1 mM PMSF). Soluble TPH1 was purified by affinity chromatography with Ni-NTA resin with elution buffer containing 300 mM imidazole (washing buffer consisting of 50 mM Tris pH 8.0, 400 mM NaCl, 3 mM methionine, 5% glycerol, and 20 mM imidazole). The protein was further purified using a Superdex 200 Increase column (GE Healthcare) equilibrated with 50 mM Tris pH 8.0, 200 mM NaCl, 0.5 mM TCEP, and 5% glycerol. The human TPH2 gene was cloned into the pETM-41 vector between restriction sites *Nco*I and *Kpn*I and overexpressed at 37 °C overnight using BL21(DE3) cells in the LB medium with 50 μg/mL kanamycin. The cell pellet was lysed through sonication in 20 mM Na-Hepes pH 7.0, 20% glycerol, 1 mM PMSF, EDTA-free protease inhibitor cocktail. Soluble TPH2 was purified by affinity chromatography with amylose-resin with elution buffer containing 10 mM maltose (washing buffer consisting of 15 mM Na-Hepes, 0.2 M NaCl, 1 mM EDTA, 1 mM DTT, and 10% glycerol). TEV protease was used to cut-off the MBP-tag and the protein was further purified using a Superdex 200 Increase column (GE Healthcare) equilibrated with 20 mM Na-Hepes, 200 mM NaCl, 10% glycerol, pH 7.0.

TH1 was expressed and purified as described.^52,53^ Human TH1 cDNA was cloned into the pET3a expression vector^22^ and expressed in *E. coli* BL21(DE3) pLysS. Single colonies were inoculated into LB medium containing 50 μg/mL ampicillin and 34 μg/mL chloramphenicol and grown overnight at 30 °C. Bacteria were grown at 37 °C in 1 L of the antibiotic-containing LB medium until OD_600_ was 0.6. Cells were disrupted using a French pressure cell. The lysate supernatant was applied to the heparin Sepharose column pre-equilibrated with 4× CV equilibration buffer (20 mM Tris-HCl pH 8.2, 150 mM NaCl, 1 mM EDTA, 1 mM DTT, 5% sucrose, 1 mM benzamidine, 0.25 mM PMSF). After binding, the column was washed with the same buffer before TH1 was eluted with stepwise increasing NaCl gradient via the combination of equilibration and elution buffers. TH1-containing fractions were pooled, concentrated, and the proteins were further purified by size-exclusion chromatography (SEC) using a Superdex 200 Increase 10/300 GL column (GE Healthcare). The SEC buffer contained 20 mM Na-Hepes pH 7.4, 200 mM NaCl and 1 mM DTT. TH1 was also expressed in BL21, CodonPlus Competent Cells (Agilent) as a His-tagged ZZ-fusion protein (His_6_-TH1) from the pET-ZZ-1a vector.^53^ His_6_-TH1 was expressed at 28 °C in autoinduction medium containing 100 μg/mL kanamycin. Bacteria were harvested by centrifugation (4000 rpm, 20 min, 4 °C), and the pellets were kept at −80 °C until further use. Purification of TH1 from bacterial pellets was performed as follows: pellets were resuspended in 50 mM Na-phosphate, 300 mM NaCl, pH 7.0 for purification on TALON metal affinity columns (Clontech) containing 1 mM PMSF. The cells were disrupted by sonication (Vibra-Cell, Sonics & Materials, Inc.), and clarified extract was applied to the resin. The His_6_-TH1 fusion protein was eluted using buffer supplemented with 150 mM imidazole and concentrated with 50 kDa cut-off Amicon Ultra Centrifugal filters (Millipore Corporation). Imidazole was removed using PD-10 columns (GE Healthcare) before the fusion protein was cut using His-tagged TEV. To remove the ZZ-fusion partner and TEV from TH1, the sample was applied to a second TALON column, leaving pure TH1 in 20 mM Na-Hepes and 200 mM NaCl, pH 7.0.

Human PAH cDNA was cloned into the pMAL expression vector, expressed in *E. coli* and purified as fusion proteins with maltose-binding protein and a linker region containing a recognition site for the serine protease factor Xa.^54^ Overnight cultures of TB1 cells were inoculated into 1 L of LB medium containing 50 μg/mL of ampicillin. Cells were grown at 37 °C and expression of hPAH was induced by the addition of 1 mM IPTG when OD_600_ was about 0.8. Ferrous ammonium sulfate (0.2 mM) was added to the medium, bacteria were harvested 18–21 h after induction, and the pellets were resuspended in medium containing 10 mM Tris/HCl, 0.2 M NaCl, 0.2 mM PMSF, 1 mM EDTA, 10 mM benzamidine, and 10% (w/v) glycerol, pH 7.4, and disrupted by passage through a French press (type FA-073 from SLM Instruments). The lysate was diluted in 10 mM Tris/HCI, 0.2 M NaCl, 0.2 mM PMSF, and 1 mM EDTA, pH 7.4, to reduce the protein concentration to about 3 mg/mL before application to the column (2.5 cm × 10 cm) of amylose resin (New England Biolabs), equilibrated with 10 mM Tris/HCl, 0.2 M NaCl and 1 mM EDTA, pH 7.4. The column was washed with about 40 CV of the equilibration buffer, and the fusion protein was eluted with buffer containing 10 mM maltose. The fusion protein was further cleaved by incubation with factor X1 for 12–16 h at 0 °C pure tetrameric PAH was obtained by gel filtration on a Superdex-75 column equilibrated with 20 mM Na-Hepes pH 7.0, containing 200 mM NaCl.^54^

### Differential Scanning Fluorimetry

A fluorescence-based thermal stability assay (differential scanning fluorimetry, DSF) was used for testing the effects of HDIs on the thermal stability of TPH1.

For initial high throughput screening (HTS), compounds were prepared at a concentration of 10 mM (in some cases, 5 mM) in 100% DMSO. Initial HTS screening was performed on TPH1 with and without compounds using a LightCycler 480 Real-Time PCR System (Roche Applied Science). TPH1 was diluted to 0.075 mg/mL in 50 mM Tris pH 8.0, 200 mM NaCl, 0.5 mM TCEP, and 5% glycerol with 5x SYPRO Orange. Compounds were added to a final concentration of 50 μM. Controls with 0.5% DMSO were performed on each 384-well microplate. Unfolding curves were recorded from 20 to 95 °C at a scan rate of 2 °C/min and monitored at excitation = 465 nm and emission = 610 nm. Selected hits were validated further by serial dilution from 250 μM to 122 nM, keeping the DMSO concentration constant at 2%.

DSF data were analyzed using HTSDSF Explorer.^55^ Melting temperature (*T*_m_) was calculated from the maximum value of the first derivative of the unfolding curve. Δ*T*_m_ (*T*_m_ with compound–averaged *T*_m_ in DMSO controls) was plotted for all validated curves and a threshold of 2 °C was set (typically, cutoff at Δ*T*_m_ = 3-fold the SD of the *T*_m_ of the DMSO control).

Preliminary binding constants (*K*_D_) were calculated by fitting data to the equation
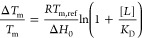
where Δ*H*_0_ is the enthalpy of the unfolding of the protein in the presence of ligand ([*L*]), and *R* is the gas constant.

### Enzyme Activity Measurements

The activity of purified PAH, TH, TPH1, and TPH2 was measured using high-performance liquid chromatography (HPLC) as previously described.^50,56,57^

TPH1 enzymatic activity assays were performed with the selected hits from the DSF screening in the presence and absence of 50 μM of each compound. The final concentration of DMSO was 0.5% (v/v), and control experiments used 0.5% DMSO in the absence of ligand. Assays were performed in a standard reaction mixture containing 40 mM Hepes pH 7.0, 200 mM NaCl, 0.1 mg/mL catalase, 0.5 mg/mL BSA, 10 μM ferrous ammonium sulfate, and 30 μM l-tryptophan (l-Trp). Compounds were incubated with TPH1 (0.02 mg/mL) in the reaction mixture for 10 min on ice. The enzymatic reaction was initiated by adding 200 μM BH_4_ and 2 mM DTT (final concentrations) and stopped by precipitation with 2% (v/v) acetic acid in ethanol after 3 min at 37 °C. The product, 5-hydroxy-tryptophan (5-OH-Trp), was quantified by HPLC utilizing a fluorescence monitor set at 302 and 350 nm for excitation and emission, respectively. Compounds that reduced the TPH1 activity by more than 50% in the preliminary activity assay were selected for further dose–response analyses (0–50 μM). Activity for PAH and TH was also tested at this point.

PAH activities were performed in a standard reaction mixture containing 100 mM Hepes (pH 7.0), 0.04 mg/mL catalase, 0.05% BSA, 10 μM ferrous ammonium sulfate, and 1 mM phenylalanine (Phe). Compounds were preincubated with PAH (0.5 μg/mL) in the reaction mix for 5 min at 37 °C. The enzymatic reaction was initiated by adding 75 μM BH_4_ and 2 mM DTT (final concentrations) and stopped by precipitation with 2% (v/v) acetic acid in ethanol after 1 min incubation. The product, l-Tyrosine (l-Tyr), was quantified by HPLC utilizing a fluorescence monitor set at 274 and 304 nm for excitation and emission, respectively.

TH activities were performed in a standard reaction mixture containing 40 mM Hepes (pH 7.0), 0.1 mg/mL catalase, 10 μM ferrous ammonium sulfate, and 50 μM l-Tyr. Compounds were incubated with TH (0.01 mg/mL) in the reaction mixture for 10 min on ice. The enzymatic reaction was initiated by adding 200 μM BH_4_ and 2 mM DTT (final concentrations) and stopped by precipitation with 2% (v/v) acetic acid in ethanol after 5 min at 37 °C. The product, L-DOPA, was quantified by HPLC utilizing a fluorescence monitor set at 281 and 314 nm for excitation and emission, respectively.

GraphPad Prism (version 10; La Jolla, CA) was used for analyzing the enzyme inhibition data. The following equation was fitted to the data using nonlinear regression, yielding an estimate of the IC_50_:
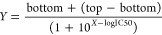
where *Y* is the response as a fraction of 1, *X* is the logarithm of ligand concentration, top is the maximum response, and bottom is the minimum response in the presence of ligand.

Steady-state kinetic studies were performed to explore the mode of inhibition. Kinetic protein–ligand interaction for TPH1 was investigated on EG22 and for TH using panobinostat. TPH1 and TH activity assays were performed as indicated above in the absence and presence of three different compound concentrations (TPH1: 0–100 μM; TH: 0–25 μM). Product formation was measured either at a fixed concentration of the cofactor BH_4_ (200 μM) and varying concentrations of l-Trp (0.937–60 μM) or l-Tyr (3.125–50 μM), respectively, or in the presence of varying concentrations of BH_4_ (TPH1:12.5–400 μM; TH: 7.4–200 μM) and a fixed concentration of amino acid substrates: 30 μM of l-Trp or 50 μM of l-Tyr, respectively. Kinetic parameters were obtained from the Michaelis–Menten model using nonlinear regression fit
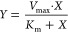
where *V*_max_ is the maximum enzyme velocity and *K*_m_ the Michaelis–Menten constant.

Lineweaver–Burk plots were obtained by plotting the reciprocal of the rate of the enzyme reaction (1/*V*) versus the reciprocal substrate concentration (1/[*S*]) in the absence and presence of compounds.

### HDAC1 Activity Assay

HDAC1 inhibition was determined by using a homogeneous fluorescence assay as previously described.^58^ For HDAC1 activity assay, OptiPlate-96 black microplates (PerkinElmer) were used. Assay volume was 60 μL, and 52 μL of human recombinant HDAC1 (BPS Bioscience, catalog no. 50051) in incubation buffer (50 mM Tris-HCl, pH 8.0, 137 mM NaCl, 2.7 mM KCl, 1 mM MgCl_2_, and 1 mg/mL BSA) were incubated with increasing concentrations of inhibitors in DMSO and 5 μL of the fluorogenic substrate ZMAL (Z-(Ac)Lys-AMC) (126 μM) for 90 min at 37 °C. After incubation time, 60 μL of the stop solution (5 μL trichostatin A (TSA) (33 μM) and 10 μL trypsin (6 mg/mL) in trypsin buffer (Tris-HCl 50 mM, pH 8.0, NaCl 100 mM)) was added. The plate was incubated again at 37 °C for 30 min, and fluorescence was measured on a BMG LABTECH FLUOstar OPTIMA plate reader (BMG Labtechnologies) with an excitation wavelength of 390 nm and an emission wavelength of 460 nm. IC_50_ values for panobinostat were determined using GraphPad Prism 9.0.2.

### Cell Culture

HepG2 cells were cultured in DMEM with high glucose supplied with 10% fetal calf serum, penicillin/streptomycin, and glutamine. The medium was changed every 2 days, and the cells were passaged as soon as they reached 85% confluence.

### Cellular Thermal Shift Assay

For CETSA, HepG2 cells were incubated with 50 μM TB57, 50 μM panobinostat, or 0.5% DMSO for 1 h. Then, cells were washed, harvested using trypsin, and pelleted by centrifugation. The pellet was washed twice with PBS supplemented with complete EDTA-free protease inhibitor cocktail (Roche) and divided into 14 aliquots of 35 μL containing 800,000 cells. Cells were then frozen in liquid nitrogen and thawed at 25 °C three times, and each aliquot was heated to a defined temperature by applying a gradient between 39 °C and 69 °C in a thermocycler (peQSTAR, PeQlab; 16-well gradient, 54 °C ± 15 °C). After 3 min incubation, aliquots were snap frozen in liquid nitrogen and thawed at 25 °C. Cell lysates were centrifuged at 20,000 *g* (4 °C) for 20 min. Supernatants were mixed with 5x loading buffer, heated to 70 °C for 5 min and analyzed by Western blotting. Chemoluminescence signals were recorded with a FusionSL, Vilber Lourmat (peQlab) and quantified using FusionCapt Advance FX7 16.01c software. Calculation of Δ*T*_m_ was conducted using the Boltzmann sigmoidal model in GraphPad prism 7.01.

### Western Blot Analysis

Cell lysates used for Western blot analysis were prepared as described in the Methods section (Cellular Thermal Shift Assay). For Western blot, anti-PAH (#191415, lot GR227200-9, Abcam/1:1000,) 1 antibody and antirabbit, HRP conjugated (#A9169, lot.: 86280 Sigma) 2 antibody were used. Chemoluminescence signals were recorded with an FusionSL, Vilber Lourmat (peQlab) and quantified using FusionCapt Advance FX7 16.01c software.

### Ligand Docking

The available X-ray structures of PAH (PDB ID: 3PAH), TPH1 (PDB ID: 8CJM), and TH (PDB ID: 6ZN2) were downloaded from the Protein Data Bank (PDB; www.rcsb.org). When analyzing all available PAH crystal structures, it could be observed that two conserved water molecules reside nearby the catalytic zinc ion. A similar observation could not be made for crystal structures of TPH and TH. Hence, only the conserved water molecules were retained in the crystal structure of PAH.

Protein preparation was performed using the protein preparation wizard in Schrödinger (Schrödinger Release 2021-3: Protein Preparation Wizard; Epik, Schrödinger, LLC, New York, NY, 2021; Impact, Schrödinger, LLC, New York, NY; Prime, Schrödinger, LLC, New York, NY, 2021) by adding hydrogen atoms, assigning the protonation states, and minimizing the protein using the OPLS4 force field. Ligands structures were generated, and the ligands were subsequently prepared for docking using the LigPrep tool (Schrödinger Release 2021-3: LigPrep, Schrödinger, LLC, New York, NY, 2021), and energy was minimized using the OPLS4 force field. The hydroxamate form of the ligands was kept for further docking studies. Twenty-five conformers of all ligands were subsequently generated using ConfGen (Schrödinger Release 2021-3: ConfGen, Schrödinger, LLC, New York, NY, 2021). Docking of the generated conformers into the prepared protein structures was performed using Glide (Schrödinger Release 2021-3: Glide, Schrödinger, LLC, New York, NY, 2021) in the Standard Precision (SP) mode.^59^

Top-ranked docking complexes were visualized using PyMOL (PyMOL Molecular Graphics System, Version 1.8.4.0 Schrödinger, LLC).

### Docking Validation

Redocking was performed in the crystal structure of PAH (3PAH); an RMSD of 1.3 Å was obtained. Meanwhile, redocking in the used TPH1 crystal structure (PDB ID: 8CJM) yielded an RMSD of 0.8 Å between the obtained docking pose and the cocrystallized ligand. Redocking in TH was not performed for the following reasons: only two available structures of TH in complex with a ligand, namely, dopamine, are deposited in the PDB (PDB ID: 6ZN2 and 7PIM). These structures show a bad resolution of ca. 4.6 Å and the exact position; i.e., binding mode, of the ligand is unclear. The binding modes of dopamine in both structures show an RMSD of 4.0 Å.

## Supporting Information Available

Details on the compounds (Table S1), TPH1 primary screening with panobinostat, TB57, and β-thujaplicin, (Figure S1), effect on the *T*_m_ of TPH1 in the presence of panobinostat, β-thujaplicin, and negative controls (Figure S2), additional activity measurements of AAAHs in the presence of telotristat, L-PCPA, mocetinostat, vorinostat, and KV103 (Figure S3), kinetic study of TH inhibition by panobinostat (Figure S4) and its kinetic parameters (Table S2), effect of methylated hydroxamic acids on TPH1 and HDAC1 activity (Figure S5), and a table summarizing the subtype selectivities of HDIs on HDAC isoforms and AAAHs (Table S3). (PDF)

## Abbreviations

AAAHs: aromatic amino acid hydroxylases
BH_4_: tetrahydrobiopterin
CETSA: cellular thermal shift assay
DSF: differential scanning fluorimetry
HDAC: histone deacetylase
HDI: histone deacetylase inhibitor
HTS: high throughput screening
PAH: phenylalanine hydroxylase
TH: tyrosine hydroxylase
TPH: tryptophan hydroxylase
