# Supplementary material for: Aromatic Amino Acid Hydroxylases as Off-Targets of Histone Deacetylase Inhibitors
Source: ACS Chem Neurosci. 2024 Nov 11;15(22):4143–55. doi: 10.1021/acschemneuro.4c00346 (PMC11587510; doi:10.1021/acschemneuro.4c00346)
Supplement: Supplementary file 1 — cn4c00346_si_001.pdf [file cn4c00346_si_001.pdf]

Supplementary Information for the original article “Aromatic amino acid hydroxylases as off-targets of histone deacetylase inhibitors”
Anne Baumann, Niklas Papenkordt, Dina Robaa, Peter D. Szigetvari, Anja Vogelmann, Franz Bracher, Wolfgang Sippl, Manfred Jung, Jan Haavik*

Anne Baumann (Anne.Baumann@uib.no)
Department of Biomedicine, University of Bergen, 5007 Bergen, Norway

Niklas Papenkordt (Niklas.papenkordt@pharmazie.uni-freiburg.de)
Institute of Pharmaceutical Sciences, University of Freiburg, 79104 Freiburg, Germany

Dina Robaa (dina.robaa@pharmazie.uni-halle.de)
Institute of Pharmacy, Martin-Luther University of Halle−Wittenberg, 06120 Halle/Saale, Germany

Peter D. Szigetvari (Peter.Szigetvari@uib.no)
Department of Biomedicine, University of Bergen, 5007 Bergen, Norway
Division of Psychiatry, Haukeland University Hospital, 5009 Bergen, Norway

Anja Vogelmann (vogelmann.anja@googlemail.com)
Institute of Pharmaceutical Sciences, University of Freiburg, 79104 Freiburg, Germany

Franz Bracher (franz.bracher@cup.uni-muenchen.de)
Department of Pharmacy - Center for Drug Research, Ludwig-Maximilians University Munich, 81377 Munich, Germany

Wolfgang Sippl (wolfgang.sippl@pharmazie.uni-halle.de)
Institute of Pharmacy, Martin-Luther University of Halle−Wittenberg, 06120 Halle/Saale, Germany

Manfred Jung (Manfred.jung@pharmazie.uni-freiburg.de)
Institute of Pharmaceutical Sciences, University of Freiburg, Albertstr. 25, 79104 Freiburg, Germany

*Jan Haavik (Jan.Haavik@uib.no)
Department of Biomedicine, University of Bergen, 5007 Bergen, Norway
Bergen Center for Brain Plasticity, Division of Psychiatry, Haukeland University Hospital, 5009 Bergen, Norway.

Aromatic amino acid hydroxylases as off-targets of histone deacetylase inhibitors

Anne Baumann, Niklas Papenkordt, Dina Robaa, Peter D. Szigetvari, Anja Vogelmann, Franz Bracher, Wolfgang Sippl, Manfred Jung, Jan Haavik*

AUTHOR INFORMATION

Corresponding Author:

Jan Haavik- *Department of Biomedicine, University of Bergen, Norway;*

Email: jan.haavik@uib.no

ORCID

Jung, Prof. Dr. Manfred	0000-0002-6361-7716
Vogelmann, Anja	0000-0001-5266-0295
Papenkordt, Niklas	0000-0003-3420-0695
Haavik, Jan	0000-0001-7865-2808
Bracher, Franz	0000-0003-0009-8629
Szigetvari, Peter D.	0000-0002-1821-2779
Baumann, Anne	0009-0006-9386-6824
Robaa, Dina	0000-0003-4297-8130

Table S1: ID, chemical name, structure and molecular weight of the selected compounds.

ID	Chemical Name /	Structure	Molecular weight (Da)	Reference	Publication ID
EG22	4-Chloro-3-(4-chlorophenylcarbamoyl)-*N*-hydroxybenzamide / $C_{14}H_{10}Cl_2N_2O_2$	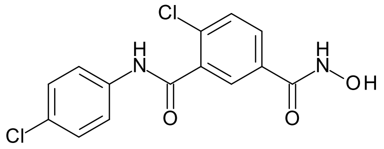	309.15	[4] E. Ghazy *et al. (*2021)	13b
EG23	3-(4-Chlorophenylcarbamoyl)-N-hydroxy-4-methylbenzamide / $C_{15}H_{13}ClN_2O_2$	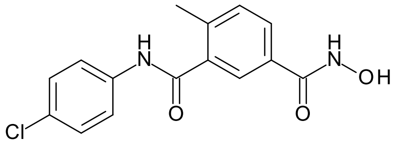	288.73	[4] E. Ghazy *et al* (2021)	13c
EG29	4-Chloro-3-[(1,3-dimethyl-2-oxo-1,2-dihydroquinolin-6-yl)sulfamoyl]-N-hydroxybenzamide / $C_{18}H_{16}ClN_3O_5S$	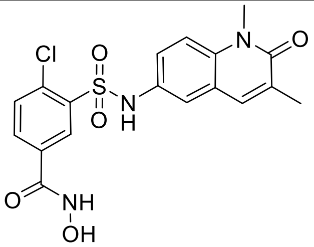	421.86	[8] E. Ghazy *et al.* (2020)	23b
JS32	2-(diphenylmethyl)-1,3-oxazole-4-carbohydroxamic acid / $C_{17}H_{18}N_2O_3$	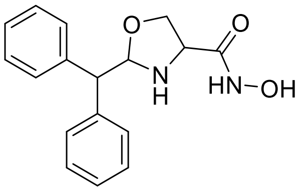	298.34	[9] J. Senger *et al. (*2016)	4i
KV46	4-[(10H-phenothiazin-10-yl)methyl]-N-hydroxybenzamide / $C_{20}H_{15}N_2O_2S$	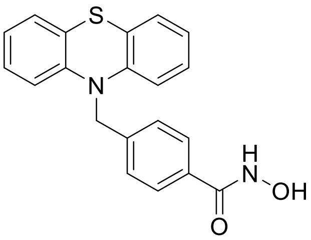	347.41	[5] K. Vögerl *et al.* (2019)	1a
KV103	N-Hydroxy-4-[(2-methoxy-10H-Phenothiazin-10-yl)methyl]benzamide / $C_{21}H_{17}N_2O_3S$	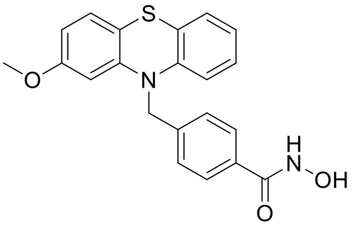	377.44	[5] K. Vögerl *et al.* (2019)	7g
SW55	$C_{24}H_{29}N_2O_5$	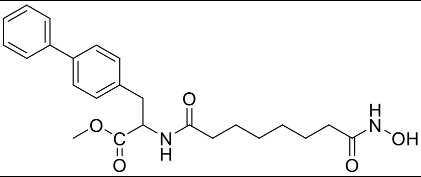	425.50	[10] S. Wittich *et al.* (2005)	BIP1
SW189	$C_{25}H_{32}N_2O_5$	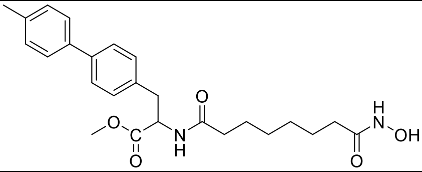	440.53	[10] S. Wittich *et al.* (2005)	BIP4

TB54	(2E)-N-Hydroxy-3-(3-methoxyphenyl)-2-propenamide / $C_{10}H_{11}NO_3$	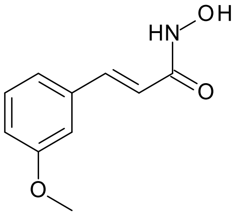	193.20	[6] T. Bayer *et al.* (2018)	19c
TB57	(2E)-3-[2-(4-Chlorophenoxy)phenyl]-N-hydroxy-2-propenamide / $C_{15}H_{12}ClNO_3$	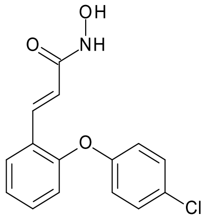	289.71	[6] T. Bayer *et al.* (2018)	32c
TB74	1H-Indole-2-hydroxamic acid / $C_9H_8N_2O_2$	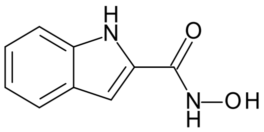	176.17	[6] T. Bayer *et al.* (2018)	11b
TH28	3-N-Benzylamino-benzohydroxamate / $C_{14}H_{15}N_2O_2$	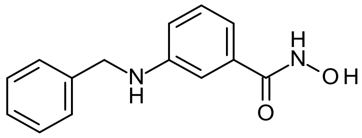	243.28	[11] T. Heimburg *et al.* (2016)	10a
TH71	3-(N-para-touluenyl)-sulfonamido-4-methoxy-benzohydroxamate / $C_{15}H_{17}N_2O_5S$	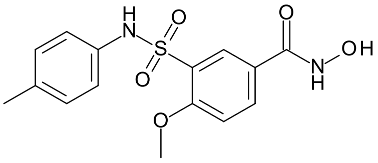	337.37	[11] T. Heimburg *et al.* (2016)	16a
PZ031	3-Benzyloxy-4-methoxybenzhydroxamic acid / $C_{15}H_{16}NO_4$	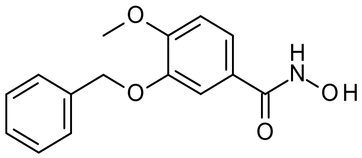	274.29	[12] T. Heimburg *et al.* (2017)	20a
AK317	$C_{21}H_{18}N_2O_1S$	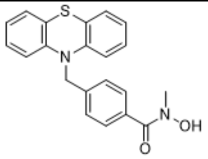	362.45		
KV-MM-04	4-[(10H-Phenothiazin-10-yl)methyl]-N-methoxy-N-methylbenzamide / $C_{22}H_{20}N_2O_2S$	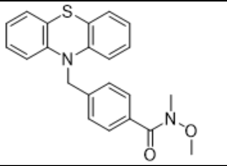	376.47	[5] K. Vögerl *et al.* (2019)	23
2-(2-hydroxyphenyl) benzoxazole	$C_{13}H_9NO_2$	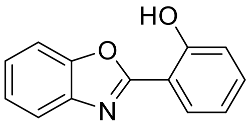	211.22		
Mocetinostat	$C_{23}H_{20}N_6O$	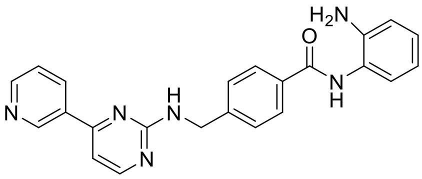	396.4		

Vorinostat	$C_{14}H_{20}N_2O_3$	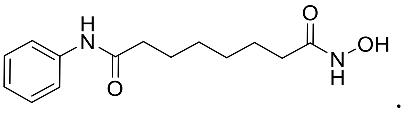	264.32		
Entinostat	$C_{21}H_{20}N_4O_3$	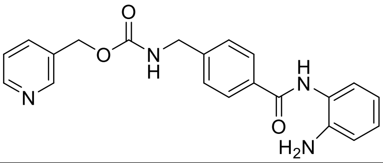	376.4		
Panobinostat	$C_{21}H_{23}N_3O_2$	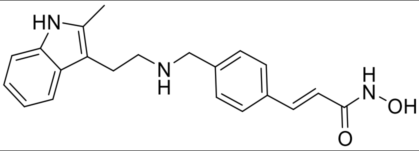	349.4		
β-Thujaplicin	$C_{10}H_{12}O_2$	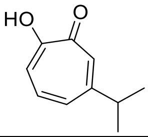	164.20		

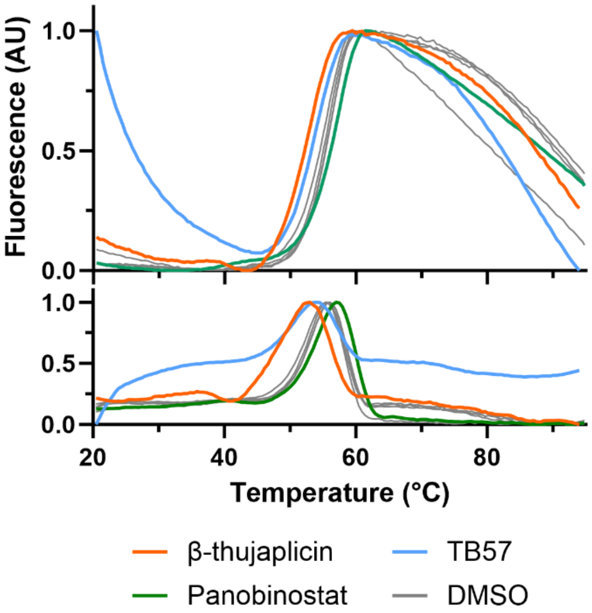

Figure S1: Thermal melting curves (upper trace) and first derivative (bottom trace) of TPH1 from primary screening monitored by DSF at 50 µM compound concentration. β-Thujaplicin and TB57 destabilize T_m of TPH1 (ΔT_m = -1.7 ± 0.1 °C and ΔT_m = -3.4 ± 0.1 °C, respectively) while Panobinostat slightly stabilizes TPH1 (ΔT_m = 0.4 ± 0.02 °C). Gray curves are 0.5% DMSO controls.

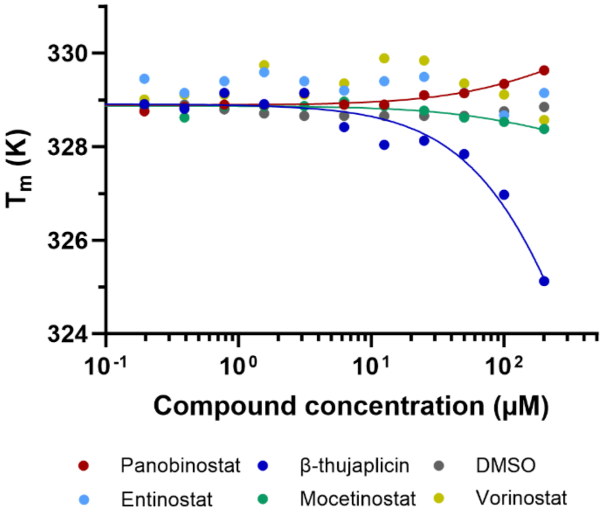

Figure S2: Effect of increasing compound concentration on the T_m of TPH1. β-Thujaplicin shows a concentration-dependent shift by decreasing the T_m of TPH1. Panobinostat (a positive control) and negative controls (mocetinostat, entinostat and vorinostat) are shown together with DMSO (solvent control).

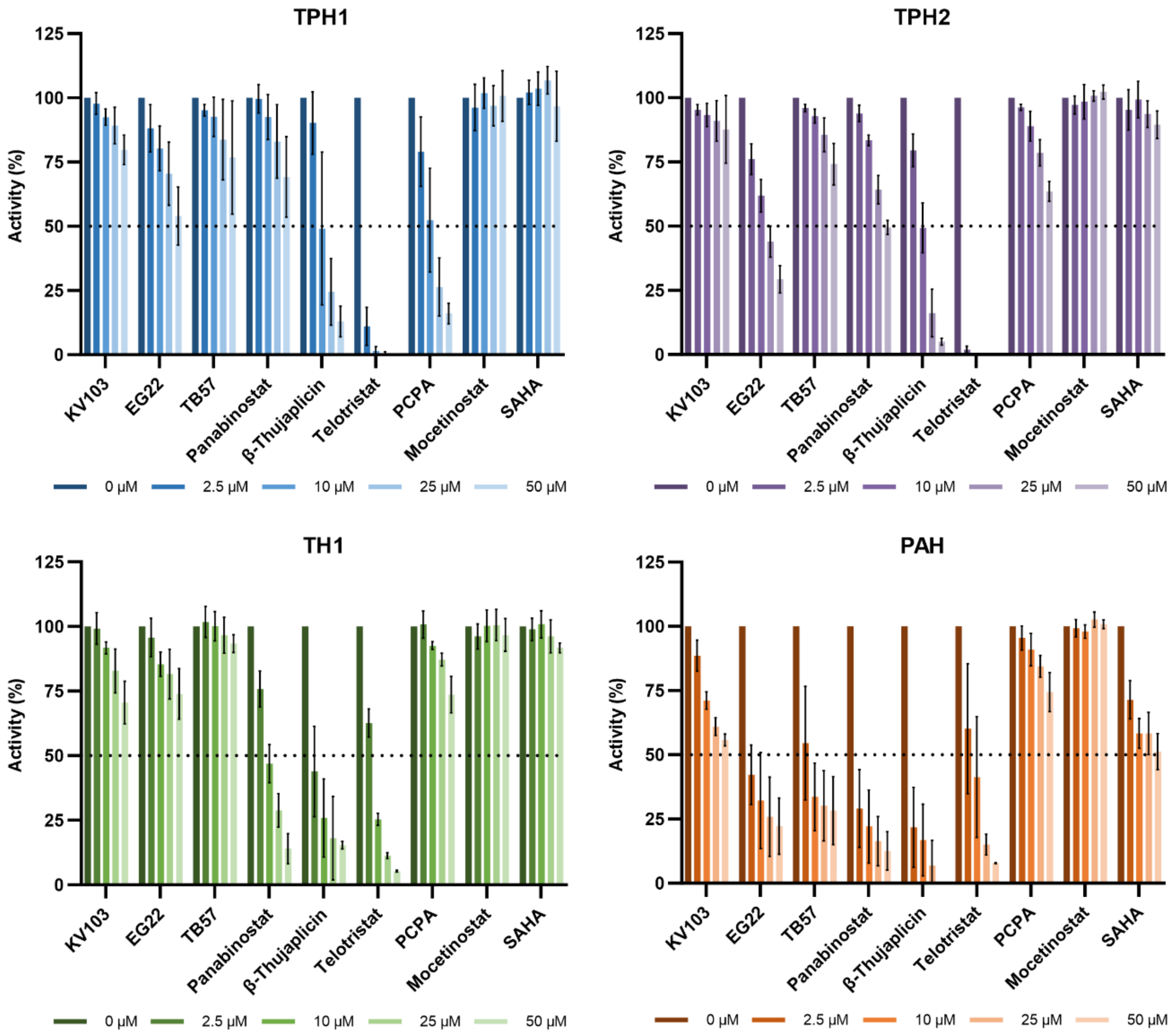

Figure S3: Dose-response of selected hits on TPH1, TPH2, TH and PAH activity (n = 4; mean ± SD).

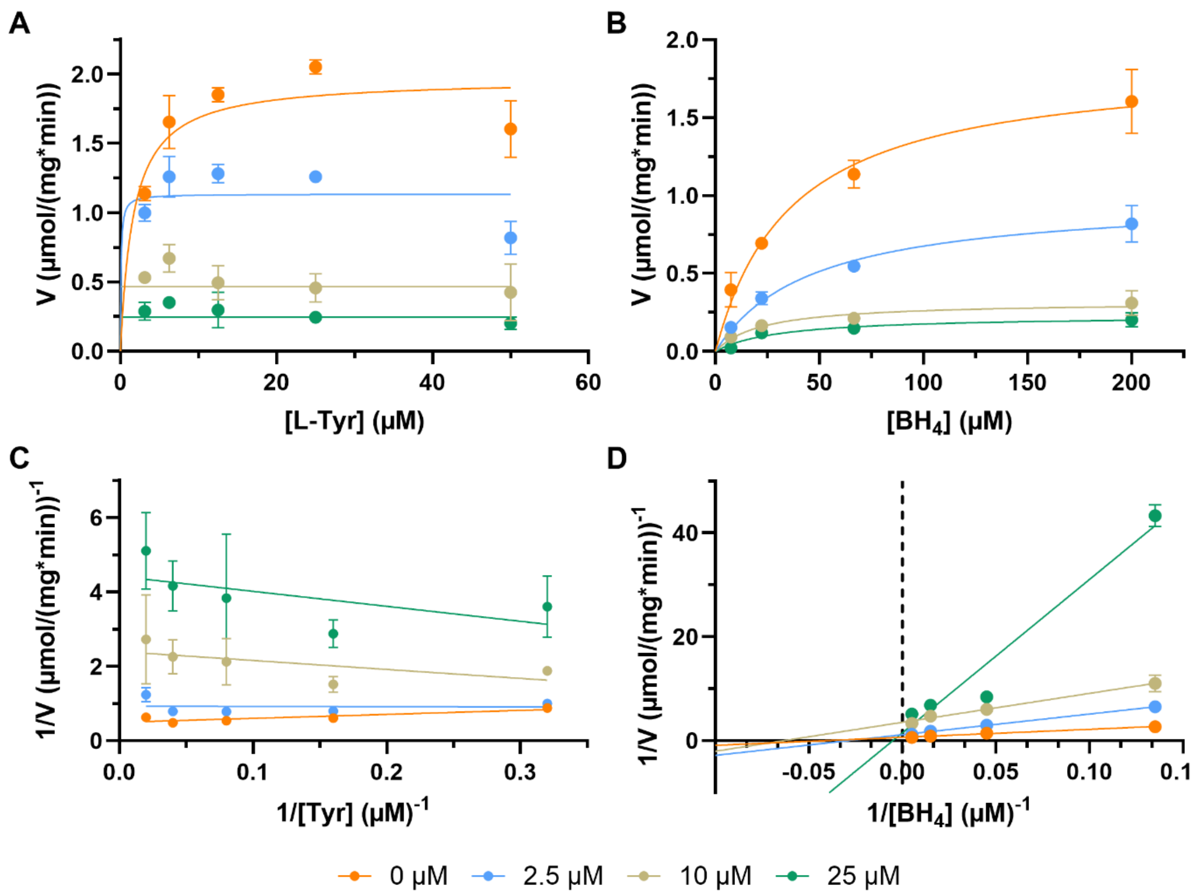

Figure S4: Kinetic study of TH inhibition by panobinostat. A-B) Michaelis-Menten equation was fitted to data using nonlinear regression. C-D) Lineweaver-Burk plots of reaction velocity versus substrate concentration for enzyme kinetics of TH at varying concentration of panobinostat (0 - 25 µM). The data represents the mean ± SD from three experiments.

Table S2: TH kinetic parameters in the presence of panobinostat. Data represents the means of three experiments. Best-fit V_{max} (± SE) and K_m (± SE) values were extracted from Michaelis-Menten nonlinear regression curve fitting.

Panobinostat (µM)	BH$_4$ 7.4 – 200 µM / L-Tyr 50 µM	
	V_{max} (µmol/(min*mg))	K_m (µM)
**0**	1.9 ± 0.1	36.5 ± 7.3
**2.5**	1.0 ± 0.1	46.2 ± 9.4
**10**	3.2E-1 ± 3.3E-2	22.3 ± 7.7
**25**	2.3E-1 ± 2.9E-2	31.0 ± 13.4

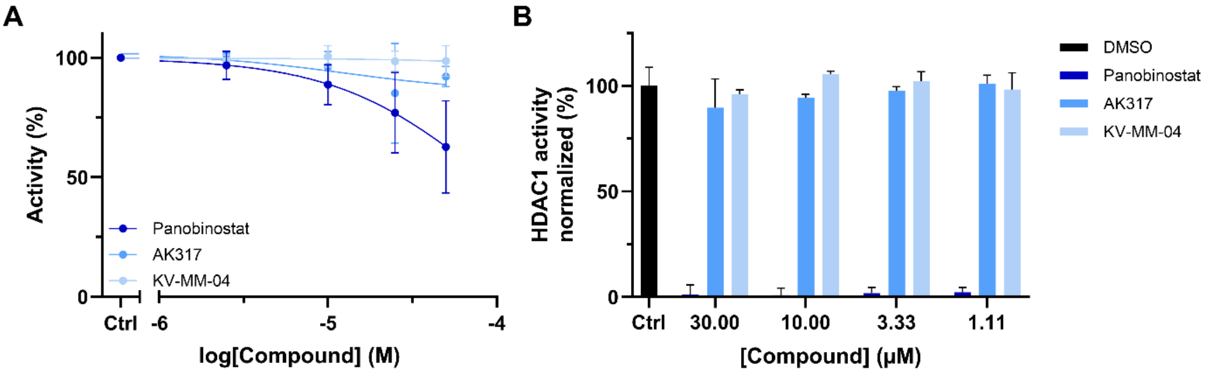

Figure S5: Effect of methylated hydroxamic acids (AK317: N-methylated; KV-MM-04: N + O methylated) on TPH1 (A) and HDAC1 (B) activity. A) The data are expressed as means ± SD from two independent experiments, each performed in duplicate. B) The data are expressed as means ± SD from triplicates.

Table S3: Activity of selected HDIs on HDAC Isoforms and AAAHs. Subtype selectivities are expressed as IC_{50} (μM) unless otherwise indicated: * IC_{50} (nM)/% inhibition; ** K_i (μM). Representative IC_{50} values were obtained through a literature search among the most recent and relevant articles indexed in PubChem. The criteria was that the authors performed original *in vitro* experiments using human recombinant HDAC isoforms. Some HDIs showed no activity (N.A.) against AAAH subtypes. Also indicated when measurements were not available from reports (no data, n.d.).

Compounds	Subtype selectivities (IC_{50}) (μM)						
	Class I HDACs		Class IIB	AAAHs			
	HDAC1	HDAC8	HDAC6	PAH	TH	TPH1	TPH2
Panobinostat	0.001 ± 0.0001[1]	0.005 ± 0.0004[1]	0.004 ± 0.0002[1]	0.57 ± 0.29	8.67 ± 1.66	95.42 ± 74.82	43.64 ± 11.53
Mocetinostat	0.098 ± 0.055[2]	35 ± 4.4[2]	>30[3]	N.A.	N.A.	N.A.	N.A.
Vorinostat	0.011 ± 0.001[1]	0.172 ± 0.045[1]	0.015 ± 0.003[1]	1.72 ± 0.61	N.A.	N.A.	N.A.
EG22	20 ± 1% @ 1 μM[4]*	0.18 ± 0.05[4]	94 ± 4% @ 1 μM[4]*	0.89 ± 0.47	14.74 ± 12.24	35.78 ± 30.96	10.72 ± 2.94
KV103	2.07 ± 0.19[5]	n.d.	0.010 ± 0.001[5]	8.12 ± 1.67	100.01 ± 128.15	106.39 ± 176.76	9.78 ± 17.20
TB57	13.4 ± 2.4[6]	0.15 ± 0.03[6]	11.4 ± 2.5[6]	1.55 ± 0.76	N.A.	49.56 ± 117.94	194.65 ± 401.08
β-thujaplicin	>2.5[7]**	0.17795[7]**	>2.5[7]**	0.46 ± 0.32	1.37 ± 0.59	12.61 ± 6.04	12.86 ± 2.58

References

1. Yang Z, Wang T, Wang F, et al. Discovery of Selective Histone Deacetylase 6 Inhibitors Using the Quinazoline as the Cap for the Treatment of Cancer. *J Med Chem*. 2016;59(4):1455-1470. doi:10.1021/acs.jmedchem.5b01342

2. Marson CM, Matthews CJ, Yiannaki E, et al. Discovery of potent, isoform-selective inhibitors of histone deacetylase containing chiral heterocyclic capping groups and a N-(2-aminophenyl)benzamide binding unit. *J Med Chem*. 2013;56(15):6156-6174. doi:10.1021/jm400634n

3. Zhou N, Moradei O, Raeppel S, et al. Discovery of N-(2-aminophenyl)-4-[(4-pyridin-3-ylpyrimidin-2-ylamino)methyl]benzamide (MGCD0103), an orally active histone deacetylase

inhibitor. *J Med Chem*. 2008;51(14):4072-4075. doi:10.1021/jm800251w

4. Ghazy E, Heimburg T, Lancelot J, et al. Synthesis, structure-activity relationships, cocrystallization and cellular characterization of novel smHDAC8 inhibitors for the treatment of schistosomiasis. *Eur J Med Chem*. 2021;225:113745. doi:10.1016/j.ejmech.2021.113745

5. Vögerl K, Ong N, Senger J, et al. Synthesis and Biological Investigation of Phenothiazine-Based Benzhydroxamic Acids as Selective Histone Deacetylase 6 Inhibitors. *J Med Chem*. 2019;62(3):1138-1166. doi:10.1021/acs.jmedchem.8b01090

6. Bayer T, Chakrabarti A, Lancelot J, et al. Synthesis, Crystallization Studies, and in vitro Characterization of Cinnamic Acid Derivatives as SmHDAC8 Inhibitors for the Treatment of Schistosomiasis. *ChemMedChem*. 2018;13(15):1517-1529. doi:https://doi.org/10.1002/cmdc.201800238

7. Ononye SN, VanHeyst MD, Oblak EZ, et al. Tropolones as lead-like natural products: the development of potent and selective histone deacetylase inhibitors. *ACS Med Chem Lett*. 2013;4(8):757-761. doi:10.1021/ml400158k

8. Ghazy E, Zeyen P, Herp D, et al. Design, synthesis, and biological evaluation of dual targeting inhibitors of histone deacetylase 6/8 and bromodomain BRPF1. *Eur J Med Chem*. 2020;200:112338. doi:https://doi.org/10.1016/j.ejmech.2020.112338

9. Senger J, Melesina J, Marek M, et al. Synthesis and Biological Investigation of Oxazole Hydroxamates as Highly Selective Histone Deacetylase 6 (HDAC6) Inhibitors. *J Med Chem*. 2016;59(4):1545-1555. doi:10.1021/acs.jmedchem.5b01493

10. Wittich S, Scherf H, Xie C, et al. Effect of inhibitors of histone deacetylase on the induction of cell differentiation in murine and human erythroleukemia cell lines. *Anticancer Drugs*. 2005;16(6). https://journals.lww.com/anti-cancerdrugs/fulltext/2005/07000/effect_of_inhibitors_of_histone_deacetylase_on_the.8.aspx

11. Heimburg T, Chakrabarti A, Lancelot J, et al. Structure-Based Design and Synthesis of Novel Inhibitors Targeting HDAC8 from Schistosoma mansoni for the Treatment of Schistosomiasis. *J Med Chem*. 2016;59(6):2423-2435. doi:10.1021/acs.jmedchem.5b01478

12. Heimburg T, Kolbinger FR, Zeyen P, et al. Structure-Based Design and Biological Characterization of Selective Histone Deacetylase 8 (HDAC8) Inhibitors with Anti-Neuroblastoma Activity. *J Med Chem*. 2017;60(24):10188-10204. doi:10.1021/acs.jmedchem.7b01447
